# Development of Wheat Lines Pyramiding the Fusarium Head Blight Resistance Gene *Fhb1* with the Stripe Rust Resistance Genes *Yr18*, *Yr28*, and *Yr36*

**DOI:** 10.3390/plants15050790

**Published:** 2026-03-04

**Authors:** Xue Yang, Peiyao Huang, Boxun Yu, Caihong Chen, Hongju Gong, Yiduo Zhang, Kebing Huang, Suizhuang Yang, Ming Hao

**Affiliations:** 1 Wheat Research Institute, College of Life Sciences and Agri-Forestry, Southwest University of Science and Technology, Mianyang 621010, China; 19166901428@163.com (X.Y.); 18881652862@163.com (P.H.); yuboxunhj@163.com (B.Y.); 15628078534@163.com (C.C.); 18166722330@163.com (H.G.); zhangyiduo@swust.edu.cn (Y.Z.); huangkebing@swust.ede.cn (K.H.); 2 Triticeae Research Institute, Sichuan Agricultural University, Chengdu 611130, China

**Keywords:** wheat, stripe rust, FHB, marker-assisted selection, pyramiding of resistance genes

## Abstract

Stripe rust (*Puccinia striiformis*) and Fusarium head blight (FHB; caused by *Fusarium graminearum*) are fungal diseases that endanger wheat productivity; however, by pyramiding disease-resistant genes, the long-term resistance of wheat can be strengthened. In this study, a multi-parent pyramiding hybrid population was constructed using marker-assisted selection (MAS). After multiple generations of breeding, 168 F_6_ lines were obtained. By combining molecular marker genotyping, field resistance identification, and agronomic trait evaluation, 19 lines with excellent agronomic traits were selected, which not only showed high resistance to stripe rust but also carried *Fhb1* genes, some of which have the potential to be developed into new germplasms and offer important genetic resources for the breeding of wheat with long-lasting and broad-spectrum resistance.

## 1. Introduction

Fusarium head blight (FHB) and stripe rust are airborne fungal diseases caused by *Fusarium graminearum* Schw. and *Puccinia striifonnis* f. sp. *tritici* (*Pst*) [1,2], respectively, that severely threaten global wheat production security. FHB causes wheat grains to become shriveled and wrinkled, reduces germination rates—thereby affecting grain quality and commercial value—and leads to the accumulation of mycotoxins such as deoxynivalenol (DON) produced by the Fusarium fungi within the grains. This, in turn, poses risks to human and animal health [3], becoming a common food security concern for major wheat-producing countries such as Europe, America, and China. In recent years, global climate change has led to frequent warm and humid weather, not only aggravating the occurrence of FHB in the wheat-growing areas of Southwest China, but also increasing the risk of its prevalence in major production areas such as North America and Europe. As an airborne disease, wheat stripe rust (*Pst*) not only inhibits leaf photosynthesis upon infection but also disrupts plant water metabolism, leading to impaired grain filling and reduced maturity [4,5]. Sichuan Province in China has become a key area for *Pst* overwintering and mutation in East Asia due to its unique topography and favorable climatic conditions. The rust population in this region exhibits a complex structure, high epidemic frequency, and broad infection range, posing a serious threat to food security both in China and globally [6,7]. As conventional chemical control methods are costly and can lead to environmental concerns, genetic improvement through the utilization of disease-resistant genes is globally recognized as the core strategy for sustainable disease management [7,8].

Resistance to FHB in wheat is a quantitative trait controlled by multiple genes [9] and can be classified into five types based on the differences in disease resistance mechanisms: type I–type V. So far, eight FHB resistance genes (*Fhb1*–*Fhb8*) have been officially named. Of these, *Fhb1*, *Fhb2*, *Fhb4*, *Fhb5*, and *Fhb8* originate from common hexaploid wheat (*Triticum aestivum*), whereas *Fhb3*, *Fhb6*, and *Fhb7* originate from wheat-related alien species [10,11]. The *Fhb1* gene, a major quantitative trait locus (QTL) conferring resistance to FHB, was originally identified in the Chinese wheat cultivar Sumai3. It is widely regarded as the most effective and stable type II FHB resistance QTL currently known in wheat [12] and was successfully cloned in 2019 by Li and Su [13,14]. Zhang et al. [12] transferred *Fhb1* to the widely cultivated variety Bannong4199 in the Huang–Huai wheat region using the diagnostic molecular markers they developed, creating materials resistant to FHB for use in regional wheat breeding. Research has found that among the hybrid offspring of the ‘Zhoumai’ varieties (lines), the materials carrying *Fhb1* have significantly better resistance to FHB than those without [15].

Stripe rust resistance genes are classified into two types: all-stage resistance (ASR) and adult-plant resistance (APR) [16]. ASR, also known as seedling resistance, can provide a high level of resistance but is usually only effective against a single pathogen species and has race specificity. Although individual ASR genes may be highly effective, they generally lack persistence and can quickly lose their efficacy due to mutations in the pathogen [17]. In contrast, some APR genes can provide broad-spectrum (usually partial) resistance that is effective against multiple *P*. *striiformis* f. sp. *tritici* races. For instance, *Yr18* [18] is a typical multi-effective disease resistance gene that can provide broad-spectrum partial resistance to leaf rust, stripe rust, stem rust, and powdery mildew which has been widely applied to numerous wheat varieties over the past few decades. *YrAS2388* (*Yr28* hereafter) [19] is an anti-stripe rust gene discovered in *Aegilops tauschii*, but not yet widely applied to wheat breeding in China. *Yr36* [20] originates from wild emmer wheat (*Triticum turgidum* ssp. *dicoccoides*) and is a non-race-specific, high-temperature-induced adult-plant broad-spectrum resistance gene. The *Yr18*, *Yr28*, and *Yr36* selected in this study are all APR genes. Studies show that the pyramiding of the three genes or even two genes can largely the adult plant resistance against wheat stripe rust but also could achieve adequate resistance in the seedling stage [19]. Taking these factors into account, integrating the major FHB resistance gene *Fhb1* with various stripe rust resistance genes is an effective approach to breeding varieties that are highly resistant and have long-lasting resistance to both diseases.

To achieve the efficient integration and precise selection of multiple disease-resistant genes, MAS has become an indispensable method that takes advantage of the close linkage between molecular markers and genes that determine target traits. By detecting molecular markers, the presence of the target genes can be identified, followed by using conventional breeding methods to screen and cultivate new varieties [17,21]. Compared with traditional breeding, MAS can greatly enhance the efficiency of gene pyramiding. Yaniv et al. [22] introduced the *Yr15* gene into wheat lines to enhance resistance to stripe rust by using a set of polymorphic SSR markers. Randhawa et al. [23] used SSR markers to introduce the stripe rust and stem rust resistance genes *Yr51*, *Yr57*, *Sr22*, *Sr26*, and *Sr50* into four wheat varieties. The continuous development and wide application of rapid and efficient molecular marker detection technologies will greatly enhance the efficiency of global breeding work.

In this study, we aim to integrate the major FHB resistance gene *Fhb1* with multiple stripe rust resistance genes (*Yr18*, *Yr28*, and *Yr36*) through MAS, using systematic molecular marker genotyping, field resistance identification, and investigation of the major agronomic traits of the constructed aggregate population’s offspring to create a new wheat germplasm that carries multiple disease-resistant genes and possesses excellent agronomic traits. This will enrich the genetic resources for FHB and stripe rust resistance in the wheat region of southwest China and provide an important material foundation for subsequent disease resistance breeding.

## 2. Results

A total of 168 F_6_ lines were selected due to their resistance to stripe rust, moderate plant height, high tillering capacity, and well-filled ears. Of these, 73 lines were selected from MY1, 18 lines were selected from MY2, and 77 lines were selected from MY3.

### 2.1. Molecular Marker Detection

**Figure 1:**
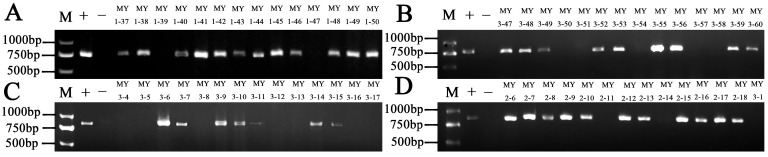
Partial molecular marker assay results for 168 F_6_ lines. M: DNA marker. +: The positive controls are Wangshuibai, Jimai22, Shumai1675, and Shumai1701. −: The negative control is Avocet S. (**A**): Amplification of wheat with the Fhb-linked marker WGRB619. (**B**): Amplification of wheat with the Yr18-linked marker L34SPF/L34DINT13R2. (**C**): Amplification of wheat with the Yr28-linked marker LG3F4/LG3R4. (**D**): Amplification of wheat with the Yr36-linked marker WKS1_150F/WKS1_620R.

The 168 lines were genotyped using functional markers or closely linked markers for the known disease-resistant genes *Fhb1*, *Yr18*, *Yr28*, and *Yr36* (Figure 1). Of these, 90 lines contained *Fhb1* (Figure 1A), 67 contained *Yr18* (Figure 1B), 46 contained *Yr28* (Figure 1C), and 71 contained *Yr36* (Figure 1D), with detection rates of 53.5%, 39.8%, 27.3%, and 42.2%, respectively. The combined analysis of these four genes indicates that there is a rich combination of disease-resistant genotypes in the population. In total, 39 (23.2%) samples carried *Fhb1* with one *Yr* gene (*Fhb1* + *Yr18*, *Fhb1* + *Yr28*, *Fhb1* + *Yr36*); 20 (11.9%) samples carried *Fhb1* with two *Yr* genes (*Fhb1* + *Yr18* + *Yr36*, *Fhb1 +Yr28 + Yr36*); 8 (4.8%) samples carried *Fhb1* with three *Yr* genes (*Fhb1* + *Yr18* + *Yr28* + *Yr36*); 16 (9.5%) samples carried two *Yr* genes (*Yr18* + *Yr28*, *Yr18* + *Yr36*); 6 (3.6%) samples carried three *Yr* genes (*Yr18* + *Yr28* + *Yr36*); 24 (14.3%) samples carried the single gene *Fhb1*; 14 (8.3%) samples carried the single gene *Yr18*; 7 (4.2%) samples carried the single gene *Yr28*; and 10 (6.0%) samples carried the single gene *Yr36*. Notably, 24 (14.3%) samples did not test positive for any of the four disease resistance genes mentioned above.

### 2.2. Stripe Rust Resistance Evaluation

Of the 168 total lines selected, 1 showed immunity to stripe rust in the field, 106 showed resistance, 41 showed intermediate resistance, and 20 were shown to be susceptible, as set out in Table 1. This shows that the 168 lines screened had overall high levels of resistance at the adult plant stage.

**Figure 2:**
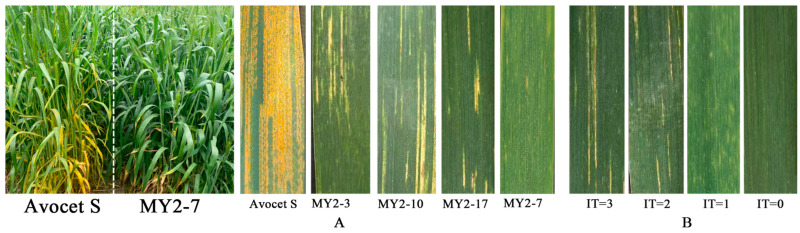
(**A**): Stripe rust resistance reactions on the leaves of the susceptible control variety (Avocet S) and MY2 plants from the F_6_ generation at the adult plant stage. (**B**): Phenotypes of the nineteen lines ultimately selected: IT = 3 (MY2-3, MY2-10, MY2-17), IT = 2 (MY1-35, MY1-55, MY3-48, MY3-72), IT = 1 (MY1-38, MY1-42, MY1-43, MY1-51, MY1-64, MY1-65, MY2-7, MY2-9, MY2-16, MY3-14, MY3-75), IT = 0 (MY1-56).

**Figure 3:**
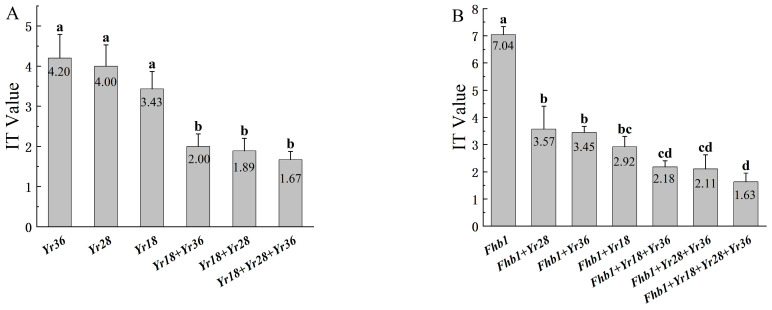
Effects of single resistance genes and their combinations on infection scores, illustrated by infection type (IT). (**A**): Stripe rust resistance phenotypes in *Yr* pyramiding populations; (**B**): stripe rust resistance phenotypes in *Yr* and *Fhb1* pyramiding populations. One-way analysis of variance was used to test the significance between each group. Lowercase letters indicate significance at *p* < 0.05, and the same letters indicate no significant difference in pairwise comparisons.

Through gene pyramiding, we developed plants harboring different combinations of the *Fhb1*, *Yr18*, *Yr28*, and *Yr36* disease resistance genes. The results are presented in Figure 2 and Figure 3. In populations combining multiple stripe rust resistance genes (Figure 3A), wheat material carrying the *Yr18* + *Yr28* + *Yr36* triple genotype exhibited high resistance in the field (IT = 1.67), as did the material carrying the *Yr18* + *Yr28* and *Yr18* + *Yr36* double genotypes (with IT values of 1.89 and 2.00, respectively). Among the single-gene wheat lines, the IT values conferred by *Yr18*, *Yr28*, and *Yr36* were 3.43, 4.00, and 4.20, respectively. Statistical analysis indicated no significant differences in stripe rust resistance within all single-gene materials and within all multi-gene materials; however, there were significant differences between single- and multi-gene materials, demonstrating that the aggregation of stripe rust resistance genes can have an enhancing effect. Among the populations with multiple rust-resistant genes, the resistance effects of each combination, ranked from highest to lowest, are as follows: *Yr18* + *Yr28* + *Yr36* > *Yr18* + *Yr28* > *Yr18* + *Yr36* > *Yr18* > *Yr28* > *Yr36*.

In the population aggregating *Fhb1* (Figure 3B), significant differences were found between the combinations of *Fhb1* + *Yr28*, *Fhb1* + *Yr36*, *Fhb1* + *Yr18* + *Yr36*, *Fhb1* + *Yr28* + *Yr36*, and *Fhb1* + *Yr18* + *Yr28* + *Yr36*. The susceptibility values of each combination, from highest to lowest, are as follows: *Fhb1* (IT = 7.04) > *Fhb1* + *Yr28* (IT = 3.57) > *Fhb1* + *Yr36* (IT = 3.45) > *Fhb1* + *Yr18* (IT = 2.92) > *Fhb1* + *Yr18* + *Yr3*6 (IT = 2.18) > *Fhb1* + *Yr28* + *Yr36* (IT = 2.11) > *Fhb1* + *Yr18* + *Yr28* + *Yr36* (IT = 1.63). This ranking is highly consistent with the ranking of the corresponding stripe rust resistance gene combinations lacking *Fhb1*. The *Fhb1* gene had no significant effect on stripe rust resistance; instead, this was primarily determined by the stripe rust resistance genes it carried, indicating no antagonistic effect between the *Fhb1* gene and the stripe rust resistance genes.

**Figure 4:**
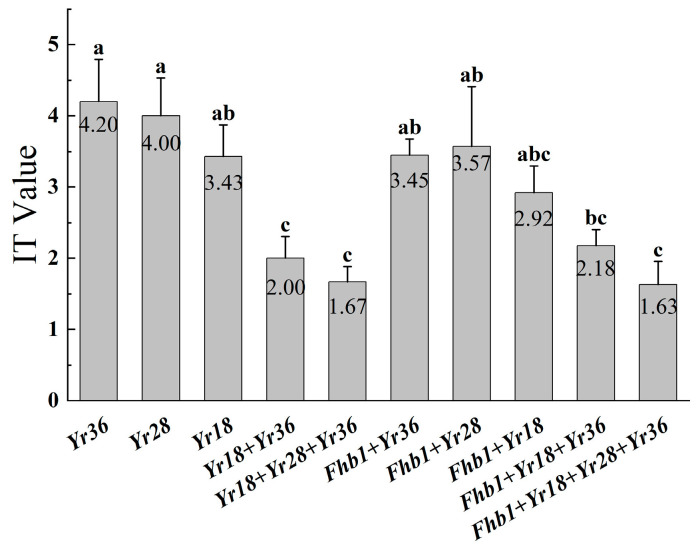
Comparison of infection types with and without the *Fhb1* gene combination. One-way analysis of variance was used to test the significance between each group. Lowercase letters indicate significance at *p* < 0.05, and the same letters indicate no significant difference in pairwise comparisons.

Infection types were compared in the combinations containing *Fhb1* and those without. As shown in Figure 4, the single-gene combinations scored the following values: *Yr36* (IT = 4.20) > *Fhb1* + *Yr36* (IT = 3.45), *Yr28* (IT = 4.00) > *Fhb1* + *Yr28* (IT = 3.57), *Yr18* (IT = 3.43) > *Fhb1* + *Yr18* (IT = 2.92). The multi-gene combinations scored as follows: *Yr18* + *Yr36* (IT = 2.00) < *Fhb1* + *Yr18* + *Yr36* (IT = 2.18), *Yr18* + *Yr28* + *Yr36* (IT = 1.67) > *Fhb1* + *Yr18* + *Yr28* + *Yr36* (IT = 1.63). The overall infection types of the combinations with *Fhb1* are lower than those without. However, statistical tests show no significant differences among the combinations.

### 2.3. Agronomic Traits Performance

**Figure 5:**
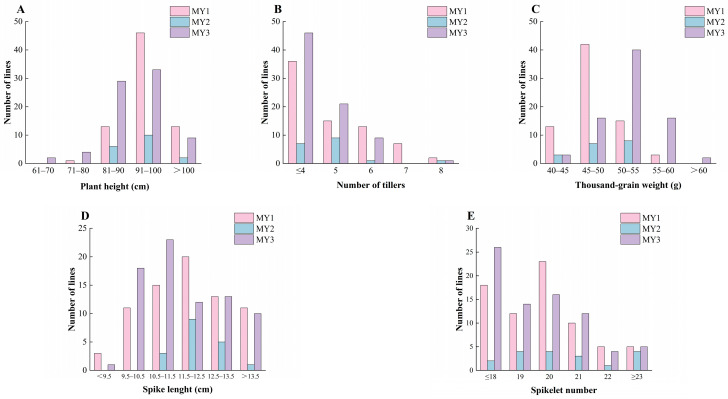
Frequency distribution of agronomic traits for the 168 lines from the three populations. (**A**) Plant height (PH); (**B**) number of tillers (NT); (**C**) thousand-grain weight (TGW); (**D**) spike length (SL); (**E**) spikelet number (SN).

At maturity, multiple agronomic traits (PH, NT, TGW, SL, and SN) of the 168 F_6_ lines were investigated (Figure 5). The mean PHs of the F_6_ generation lines from the three combinations were 95.63, 94.39, and 91.45 cm, respectively, mainly distributed in the 80–100 cm range (Figure 5A). The mean NTs of the three combinations were 5.00, 4.72, and 4.39, respectively, mainly distributed in the 4–6 range (Figure 5B). The mean TGWs of the three combinations were 47.77, 49.55, and 52.34 g, respectively, mainly distributed in the 45–55 g range (Figure 5C). The mean SLs of the three combinations were 11.91, 12.28, and 11.76 cm, respectively, mainly distributed in the 9.5–13.5 cm range (Figure 5D). The mean SNs of the three combinations were 19.53, 20.61, and 19.42, respectively, mainly distributed in the 18–21 range (Figure 5E). Overall, this demonstrates that these lines can serve as crucial intermediate materials, providing a genetic foundation for breeding new wheat varieties with different desirable agronomic traits.

### 2.4. Effects of Fhb1 and Yr Genes Pyramiding on Agronomic Traits in Plant Populations

**Figure 6:**
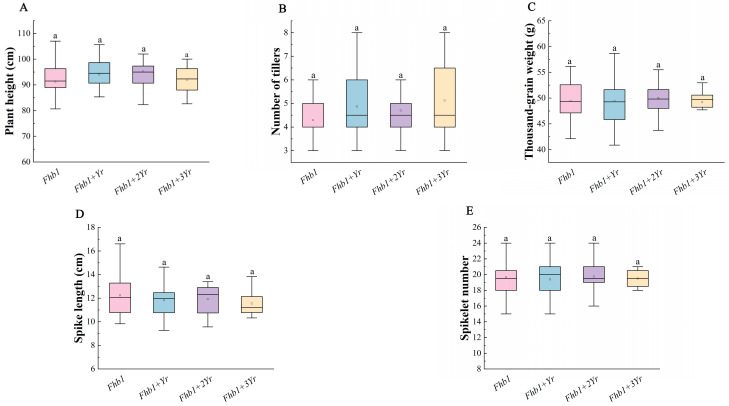
Comparison of *Fhb1* and *Yr* gene pyramiding lines’ agronomic traits in the F_6_ population. (**A**) Plant height (PH); (**B**) number of tillers (NT); (**C**) thousand-grain weight (TGW); (**D**) spike length (SL); (**E**) spikelet number (SN). One-way analysis of variance was used to test the significance between each group. Lowercase letters indicate significance at *p* < 0.05, and the same letters indicate no significant difference in pairwise comparisons.

Based on the resistance of the population combining *Fhb1* and *Yr* genes (*Yr18*/*Yr28*/*Yr36*), we further analyzed the performance of its agronomic traits. The results indicated (Figure 6) no significant differences in PH, NT, SL, SN, and TGW among the different disease-resistant gene combinations, showing that the combination of *Fhb1* and *Yr* genes does not have an impact on agronomic traits and can therefore be used to maintain both their excellent original agronomic traits and the multiple introduced resistances. This provides an important basis for breeding new superior wheat varieties with dual resistance to FHB and stripe rust and stable agronomic traits.

### 2.5. Comprehensive Screening of Superior Germplasm for Disease Resistance

The 168 F_6_ lines were screened using the standard selection criteria for wheat breeding. The selection criteria were PH ranging from 80 cm to 100 cm, PH ≥ 18, NT ≥ 4, and TGW > 45 g. This process was conducted in conjunction with the comprehensive strip rust resistance assessment. Ultimately, 19 lines were selected from the F_6_ lines (Table 2): 9 from the MY1 combination, 6 from the MY2 combination, and 4 from the MY3 combination. Of these, nine wheat germplasms carrying two pyramided resistance genes (*Fhb1* + *Yr36*, *Fhb1* + *Yr18*, and *Fhb1* + *Yr28*); eight germplasms carrying three pyramided resistance genes (*Fhb1* + *Yr18* + *Yr36* and *Fhb1* + *Yr28* + *Yr36*); and two germplasms carrying four pyramided resistance genes (*Fhb1* + *Yr18* + *Yr28* + *Yr36*) were identified.

## 3. Discussion

Wheat is one of the three major global food crops, accounting for one-fifth of the Earth’s crop cultivation area; as such, it is vital that wheat production remains uncompromised. Sichuan Province, as an important summering area, wintering area, and source of wheat stripe rust in China [24], can influence the spread of the disease to other wheat-growing areas in the country and has a significant impact on the prevalence of wheat stripe rust nationwide [25]. Because virulent physiological races of stripe rust fungus mutate rapidly, wheat varieties relying on a single disease-resistant gene are prone to losing their resistance [26]. Therefore, aggregating multiple disease-resistant genes is an effective strategy for cultivating varieties with high and long-lasting resistance [27], assisted by the development of MAS technology. For instance, by crossing the slow stripe rust line ‘Longyuan932’ with the highly resistant variety ‘Lantian15’ and through the application of molecular MAS, the genes *Yr29*, *Yr30*, and *YrZH84* were successfully combined. The resulting variety showed immunity to the highly virulent CYR33 race at the seedling stage, though susceptible to CYR34, and exhibited high resistance to the mixed races at the adult stage [28]. This indicates that gene pyramiding can enhance the resistance and durability of wheat.

The aggregation of multiple disease-resistant genes has been reported in barley, cotton, rice, wheat, and other crops [29,30]. For example, research shows that the combination of *Lr34*/*Yr18* and *Lr67*/*Yr46* can simultaneously confer durability and resistance to leaf rust, stripe rust, and powdery mildew in wheat [31]. Liu et al. [32] hybridized the main promoted varieties in the Yellow and Huai River wheat region, namely Bainong64 and Lumei21, to develop a new wheat line integrating multiple genes and conferring adult-plant resistance to multiple diseases. Huang et al. [33] utilized the *YrAS2388* KASP marker to combine the *YrAS2388*, *Yr36*, *Fhb1*, and *Pm21* genes, creating F_4_-generation multi-resistance polymer plants with different combinations of disease resistance genes. The *YrAS2388* + *Yr36* + *Fhb1* + *Pm21* polyploid exhibited high resistance (0–1) to stripe rust, FHB, and powdery mildew. Zhang et al. [12] used the wheat line NMAS022 as the donor parent and Baimai4199 as the recipient parent, and through molecular marker-assisted backcross breeding, they developed a new wheat line, Bainong4299, that integrated *Fhb1*, *Fhb4*, and *Fhb5* from Wangshuibai. Compared with Bainong4199, Bainong4299 maintained the same yield while increasing type I resistance by at least 73% to 74% and type II resistance by at least 83% to 88%. In this study, we combined molecular marker-assisted and traditional breeding methods to conduct gene pyramiding by integrating the *Fhb1* gene with the disease resistance genes *Yr18*, *Yr28*, and *Yr36*. Our field results show that the resistance levels of lines that aggregated multiple stripe rust resistance genes is higher than that of single-gene type materials. The four-gene polymeric material (*Fhb1* + *Yr18* + *Yr28* + *Yr36*) exhibited the highest resistance in field trials (IT = 1.63), demonstrating that the pyramiding of APR genes can enhance wheat resistance to stripe rust.

To further cultivate a multi-resistant germplasm with FHB resistance, we utilized *Fhb1*, currently recognized as the most potent, stable, and most frequently used type II gene for breeding resistance to FHB [12]. Pumphrey et al. [34] used molecular markers in the *Fhb1* interval to screen the US breeding population and obtained 19 near-isogenic lines (NILs) carrying *Fhb1*, which were tested with greenhouse point inoculation experiments and four field FHB resistance screening plots. The NILs carrying the *Fhb1* resistance allele showed average reductions of 23% in disease severity scores and 27% in the percentage of diseased grains at harvest, with both differences being highly significant (*p* < 0.001). Bernardo et al. [35] obtained five NILs from the American wheat variety Clark using the flanking markers of *Fhb1*. Of these, *Fhb1* was present in the four resistant NILs, but absent in the one susceptible NIL, confirming that *Fhb1* significantly enhanced resistance to FHB and reduced DON content, with a yield level comparable to that of Clark. Zhang et al. [36] confirmed that the selection of backcross progeny carrying *Fhb1* using KASP markers can significantly enhance wheat FHB resistance. Dai et al. [37] also obtained materials with significantly enhanced resistance to FHB through marker-assisted backcrossing of *Fhb1* in multiple genetic populations; the average number of diseased SNs and severity of the 161 BC_2_F_3_ lines carrying the *Fhb1* resistance allele (*Fhb1-R*) were both lower than those of the control Huaimai20, moderately susceptible to FHB, and the recurrent parent. In our populations where the *Fhb1* and *Yr18*/*Yr28*/*Yr36* genes were aggregated, the resistance levels of materials carrying the *Fhb1* gene were highly consistent with those not carrying it. Stripe rust resistance is mainly determined by the resistance genes it carries, indicating no antagonistic effect between *Fhb1* and the stripe rust resistance genes. Further comparison between combinations with and without the *Fhb1* gene revealed that the IT values of combinations containing *Fhb1* decreased slightly, but no significant differences were found. Research indicates that *Fhb1* does not negatively affect stripe rust resistance, confirming that these resistance mechanisms can function independently and can be integrated into the same genetic background.

Currently, hundreds of FHB resistance QTLs have been identified [1]; however, most QTLs effects are weak and lack low-cost molecular markers. In contrast, there are eight formally named genes with stronger resistance to FHB (*Fhb1*–*Fhb8*) [11]. The *Fhb1* selected for this study is the most potent, stable and most frequently utilized QTL for FHB resistance. Overreliance on a single major resistance gene may carry the risk of resistance failure. Future breeding efforts should systematically explore the untapped resistance QTLs in landraces (such as Sumai3 and the Yangmai series) and wild relatives (such as *Aegilops* and *Thinopyrum*); pyramiding of FHB QTLs improves wheat FHB resistance; and increase international collaboration to broaden resistance sources. For FHB, a disease of global importance, this strategy will be key to achieving its sustainable management.

The core of creating superior varieties lies in enhancing resistance while maintaining excellent agronomic traits. The 19 lines ultimately selected in this study not only exhibited high resistance to stripe rust (0–3) but also demonstrated excellent agronomic traits (PH 87.67–100.00 cm, NT 4–9.33, SL 10.60–14.33 cm, SN 18.33–22.67, and TGW 45.73–56.98 g). These lines provide a solid foundation for breeding new wheat varieties with high resistance and excellent agronomic traits.

This study successfully created 19 new wheat germplasms that exhibit stripe rust resistance with excellent agronomic traits and carry the *Fhb1* gene for FHB resistance through a multi-gene pyramiding strategy. We have not only verified the effectiveness of gene pyramiding in enhancing durability and broad-spectrum resistance, but also provided materials for the targeted breeding of multi-resistant, high-yielding, and stable wheat varieties.

## 4. Materials and Methods

### 4.1. Plant Material

The disease-resistant gene donor materials used in this study were derived from common wheat varieties Jimai22, Shumai1675, Shumai1701, and Chuanmai64R_4_. Jimai22 originates from ([Jimai22/Moro//Jimai20]/[Jimai22/98M71//Jimai20]) and contains *Yr18* [19]; it exhibited excellent agronomic traits, including high and stable yield, wide adaptability, and lodging resistance, and it exhibited moderate to slow rusting resistance to stripe rust [38]. Shumai1675 is derived from *A. tauschii* and contains *Yr28* [39]; its agronomic traits include high and stable yields, moderate plant height (approximately 91.7 cm), and high resistance to stripe rust [40]. Shumai1701 originates from tetraploid wheat PI415152 and contains *Yr36*, an elite line that confers high-temperature APR [41], and Chuanmai64R_4_ is a strain developed by introducing the *Fhb1* gene into the background of the Sichuan wheat variety Chuanmai64. This line, building upon the excellent agronomic traits of Chuanmai64 (including high and stable yield and wide adaptability), exhibits high resistance to FHB [33]. Shumai830, approved in Sichuan in 2017, is a current leading wheat variety characterized by a large flag leaf area, high TGW, and a fast grain-filling rate, and maintains high stripe rust resistance at the APR [39,42,43]. N3347, N2146, and NJY1739-2 are materials developed by the Neijiang Academy of Agricultural Sciences, which were used to construct hybrid populations with the variety Chuanmai64R_4_. The rust-susceptible control material used was Avocet S, provided by the Wheat Research Institute of Southwest University of Science and Technology. The stripe rust fungus materials used were naturally occurring in the field.

### 4.2. Cross-Combination and Field Offspring Screening

**Figure 7:**
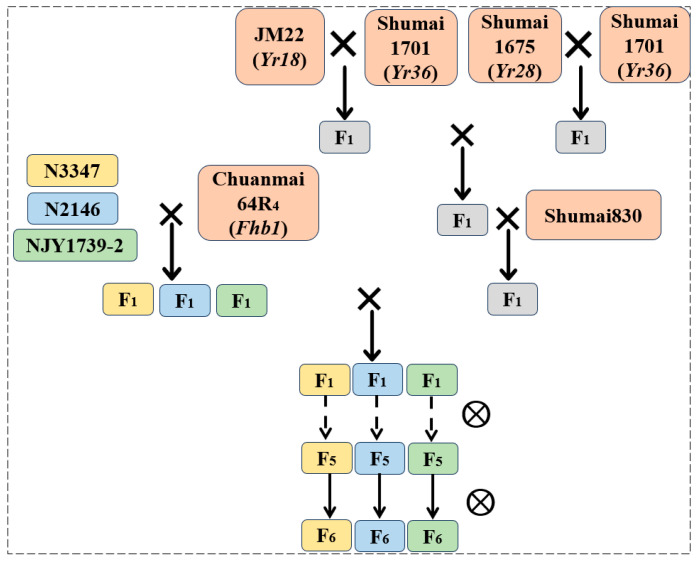
Hybridization process for multiple disease-resistant gene populations using MAS methods.

To accurately analyze the impact of gene aggregation on wheat disease resistance, Chuanmai64R_4_ (*Fhb1*) was crossed with N3347, N2146, and NJY1739-2 to construct FHB-resistant populations. Jimai22 (*Yr18*) and Shumai1675 (*Yr28*) were crossed with Shumai1701 (*Yr36*), and the F_1_ generations were crossed with each other and then backcrossed with the commercial variety Shumai830 to construct a population with multiple stripe rust resistance genes. Through crossbreeding three groups of FHB-resistant populations with multiple stripe rust-resistant populations, the resultant three sets of disease-resistant gene carrier materials were cultivated in the field in the F_1_ generation (Figure 7). In the F_2_–F_4_ generations, mass selection was carried out.

In the 2023–2024 season, F_5_ and the susceptible material Avocet S were planted in Mianyang, Sichuan Province (31°42′ N, 104°38′ E, elevation 522 m). Each cross-combination was planted in 50 rows, with a row length of 2 m, a row spacing of 30 cm, and approximately 80 seeds sown per row. Adopting local conventional field management practices, no disease control measures were taken during the planting and growing periods. In Mianyang, wheat stripe rust propagates in the winter, providing a source of fungus for spring epidemics, meaning that the experimental field was a natural source of disease. To ensure that the wheat plants in the entire field were fully infected, the susceptible variety Avocet S was planted every 20 rows. In 2024, based on agronomic trait evaluations and stripe rust resistance, a total of 168 F_6_ lines were selected from three cross-combinations.

In the 2024–2025 season, a total of 168 F_6_ lines were planted in the field. Each line was sown in three rows with a row length of 1.5 m, a row spacing of 30 cm, and approximately 30 seeds per row. The resulting disease-resistant F_6_ lines derived from the three cross-combinations were designated as MY1, MY2, and MY3 (Table 3).

### 4.3. DNA Extraction and Genotyping Wheat Lines by Markers

Plant genomic DNA was extracted from fresh leaves of the 168 F_6_ lines, employing the modified cetyltrimethylammonium bromide (CTAB) extraction method [44]. This study used functional markers to detect four known disease resistance genes. Among them, the intragenic marker primers for amplifying the *Yr28* gene are *LG3F4*/*LG3R4*, and the kinase-specific primers for amplifying the *Yr36* gene are *WKS1-150F*/*WKS1-620R*. All molecular marker primers for detecting the stripe rust (*Yr18*, *Yr28*, and *Yr36*) and FHB resistance genes (*Fhb1*) were synthesized by Sangon Biotech (Shanghai) Co., LTD. (Shanghai, China) (https://www.sangon.com) (accessed on 12 February 2025). The sequence information is shown below (Table 4). The polymerase chain reaction (PCR) was performed in a 10 uL volume, which contained 5 μL 2 × San Taq PCR Master Mix (Sangon Biotech, Shanghai, China) (with Blue Dye), 0.8 μL DNA template, 0.8 μL primer (forward and reverse primers in a volume of 0.4 μL each), and 3.4 μL ddH_2_O. The PCR amplification procedure included pre-denaturing at 94 °C for 3 min, followed by 35 cycles of denaturation at 94 °C for 30 s; annealing at 58–65 °C for 30 s (annealing temperature varied by primer); and extension at 72 °C for 30–60 s/kb. After the cycles, a final extension was performed at 72 °C for 10 min, and then the samples were kept at 10 °C for later detection. The PCR products were separated using 1% agarose gel electrophoresis, imaged with a Chemi Doc MP Universal Gel Imaging and Analysis System Imaging System, and scanned for records with Image Lab software (Version 5.0 build 18).

### 4.4. Phenotyping for Stripe Rust Reaction in the Field

In the 2024–2025 season, field resistance evaluations were conducted on 168 F_6_ lines and the susceptible control Avocet S in Mianyang, Sichuan Province. Infection type (IT) was used as the primary criterion for stripe rust resistance evaluation, scored on a 0–9 scale according to Line and Qayoum (1992) [48]: IT = 0 indicates immunity, IT = 1–3 indicates high resistance, IT = 4–6 indicates medium resistance, and IT = 7–9 indicates susceptibility. IT data were collected when the susceptible control Avocet S exhibited high susceptibility to stripe rust (IT = 7–9) and again after a week. For leaves exhibiting mixed symptoms, we assign a single IT value based on their overall appearance. In each survey, three leaves were randomly selected from each line for evaluation, and the average value was recorded. By calculating the average values between the two survey records for each line, the IT value for that line was ultimately determined.

### 4.5. FHB Resistance Evaluation

In this study, the evaluation of FHB resistance was based on genetic inference: by successfully marking the detected *Fhb1* gene and relying on a large number of previous studies that have confirmed the effectiveness of *Fhb1*, predictions were made about *Fhb1* resistance.

### 4.6. Agronomic Traits Evaluation

At maturity, agronomic traits were assessed in 168 F_6_ lines. Five single plants were randomly selected from each row to measure the following traits in the field: plant height (PH), number of tillers (NT), spike length (SL), and spikelet number (SN). After harvesting, threshing, and drying, 1000 grains were randomly selected and weighed, the process was repeated three times, and the average recorded as the thousand-grain weight (TGW). Grain length (GL), grain width (GW), and other traits were assessed using a grain measuring instrument.

### 4.7. Statistical Analysis

Statistical analysis, comparison of phenotypic data, and genetic testing results for the 168 F_6_ lines were conducted using IBM SPSS Statistics 26.

## 5. Conclusions

This study developed 19 F_6_ wheat germplasm lines through the pyramiding of the *Fhb1*, *Yr18*, *Yr28*, and *Yr36* genes. These lines exhibit high resistance to stripe rust and excellent agronomic traits, and carry the FHB resistance gene *Fhb1*. This provides valuable intermediate germplasm for the development of high-yielding, multi-resistant wheat varieties with broad-spectrum and durable resistance, as well as essential genetic resources to address the complex disease pressures in the wheat-growing regions of Southwest China.

## Figures and Tables

**Table 1:** The statistics of the number of infection type in the three cross combinations F_6_.

Cross- Combination	Number of Lines				
	Immune (IT = 0)	Resistant (IT = 1–3)	Intermediate (IT = 4–6)	Susceptible (IT = 7–9)	Total
MY1	1	40	21	11	73
MY2	0	11	5	2	18
MY3	0	55	15	7	77

**Table 2:** Stripe rust resistance and agronomic traits of the 19 lines selected.

Line	Gene	IT	Plant Height/cm	Number of Tillers	Spike Length/cm	Spikelet Number	Thousand-Grain Weight/g
MY1-35	*Fhb1* + *Yr36*	2	94.33 ± 2.03	7.00 ± 1.53	11.70 ± 0.12	21.67 ± 0.33	48.72 ± 0.97
MY1-38	*Fhb1* + *Yr18* + *Yr36*	1	96.00 ± 0.58	4.00 ± 0.58	13.33 ± 0.33	20.67 ± 0.33	55.50 ± 0.55
MY1-42	*Fhb1* + *Yr18*	1	87.67 ± 0.88	7.00 ± 2.00	13.83 ± 0.37	20.33 ± 0.67	51.20 ± 0.86
MY1-43	*Fhb1 * + *Yr36*	1	95.33 ± 1.45	6.67 ± 2.03	14.33 ± 0.60	21.00 ± 1.15	55.27 ± 1.75
MY1-51	*Fhb1* + *Yr28* + *Yr36*	1	96.33 ± 4.10	4.33 ± 0.88	12.30 ± 0.91	18.67 ± 0.88	50.73 ± 0.54
MY1-55	*Fhb1* + *Yr28* + *Yr36*	2	98.33 ± 1.33	5.67 ± 0.88	13.37 ± 0.79	19.33 ± 1.67	49.74 ± 0.31
MY1-56	*Fhb1* + *Yr18*	0	96.67 ± 2.40	6.00 ± 0.58	14.27 ± 0.46	22.00 ± 0.00	50.37 ± 0.88
MY1-64	*Fhb1* + *Yr28*	1	99.33 ± 2.40	7.33 ± 3.38	12.17 ± 0.83	20.00 ± 1.53	50.51 ± 1.44
MY1-65	*Fhb1* + *Yr36*	1	99.67 ± 3.76	7.33 ± 1.33	12.87 ± 0.81	20.00 ± 1.15	45.73 ± 0.12
MY2-3	*Fhb1* + *Yr28* + *Yr36*	3	88.67 ± 0.33	5.00 ± 0.00	13.37 ± 0.41	22.67 ± 0.33	52.46 ± 0.73
MY2-7	*Fhb1* + *Yr18* + *Yr28* + *Yr36*	1	100.00 ± 1.15	8.00 ± 1.00	11.60 ± 0.38	19.67 ± 0.33	47.72 ± 1.63
MY2-9	*Fhb1* + *Yr28* + *Yr36*	1	95.00 ± 1.15	4.67 ± 1.20	12.17 ± 0.44	19.33 ± 0.33	50.77 ± 2.21
MY2-10	*Fhb1* + *Yr36*	3	90.67 ± 3.18	5.00 ± 0.58	12.27 ± 0.27	20.00 ± 0.58	51.99 ± 1.51
MY2-16	*Fhb1* + *Yr18* + *Yr36*	1	96.00 ± 0.58	5.33 ± 0.67	12.37 ± 0.87	19.33 ± 1.20	47.34 ± 1.35
MY2-17	*Fhb1* + *Yr18* + *Yr36*	3	96.00 ± 2.52	4.00 ± 0.00	11.00 ± 0.82	18.33 ± 0.67	51.65 ± 1.47
MY3-14	*Fhb1* + *Yr28* + *Yr36*	1	95.00 ± 1.53	9.33 ± 0.88	11.43 ± 0.43	20.00 ± 0.58	48.13 ± 0.80
MY3-48	*Fhb1* + *Yr18*	2	92.00 ± 1.53	4.67 ± 0.88	11.47 ± 0.92	20.67 ± 0.33	54.88 ± 1.17
MY3-72	*Fhb1* + *Yr18*	2	89.00 ± 2.08	4.00 ± 0.58	13.10 ± 0.26	20.00 ± 0.58	56.98 ± 1.11
MY3-75	*Fhb1* + *Yr18* + *Yr28* + *Yr36*	1	92.33 ± 0.33	5.33 ± 0.33	10.60 ± 0.53	19.33 ± 0.88	52.97 ± 2.96

**Table 3:** The three hybrid combinations used.

Cross- Combination	Parental Material
MY1	(N3347×Chuanmia64R_4_)/[(Jimai22/Shumai1701 F_1_)/(Shuami1675/Shumai1701 F_1_)//Shumai830 F_7_]
MY2	(N2146×Chuanmia64R_4_)/[(Jimai22/Shumai1701 F_1_)/(Shuami1675/Shumai1701 F_1_)//Shumai830 F_7_]
MY3	(NJY1739-2×Chuanmia64R_4_)/[(Jimai22/Shumai1701 F_1_)/(Shuami1675/Shumai1701 F_1_)//Shumai830 F_7_]

**Table 4:** Molecular markers and primers used for detecting disease resistance genes in wheat.

Gene	Marker	Primer Sequence	References
*Fhb1*	*WGRB619*	F: TACTTGTGGTAGTGCCAGCTGC	[13]
		R: TCAGAGTCAGAGTGGCCATGTTTTT	
*Yr18*	*L34SPF*	F: GGGAGCATTATTTTTTTCCATCATG	[45]
	*L34DINT13R2*	R: ACTTTCCTGAAAATAATACAAGCA	
*Yr28*	*LG3F4*	F: GCACCGTCCTTCATCTCAGT	[46]
	*LG3R4*	R: TGCTTTTCCCCGTATCCCTT	
*Yr36*	*WKS1_150F*	F: ATGGAGCTCCCACGAAACAAAC	[47]
	*WKS1_620R*	R: ACCTCCATGTTGCTCGCATTTGCT	

## Data Availability

Data are contained within the article.
